# Consistently higher and steeper apparent temperature–heat-related illness risk among occupational cases in Korea: evidence from national emergency department surveillance

**DOI:** 10.3389/fpubh.2026.1786564

**Published:** 2026-03-11

**Authors:** Min Soo Park, Boo Wook Kim

**Affiliations:** Department of Occupational Health and Safety Engineering, Inje University, Gimhae, Republic of Korea

**Keywords:** apparent temperature, climate change, ED surveillance, heat-related illness, heat-risk communication, occupational vs. non-occupational, Poisson IRR

## Abstract

**Background:**

Heat-related illness (HRI) is increasing under climate change, particularly in humid regions. While heat alerts increasingly rely on apparent temperature (AT), evidence linking AT conditions to differential HRI risk by occupational status remains limited.

**Methods:**

We linked national emergency department (ED)-based HRI surveillance with daily national mean AT for June–September 2015–2024. We summarized bin-wise mean daily counts and estimated bin-specific incidence rate ratios (IRRs; reference = 24 °C) using Poisson regression.

**Results:**

HRI increased non-linearly with AT in both groups, and occupational IRRs tended to be higher than non-occupational IRRs at warmer bins. At 31 °C, occupational IRR was 37.07 vs. 23.86 for non-occupational; at 33 °C, 104.75 vs. 72.25; and at 34 °C, 167.22 vs. 141.06.

**Conclusion:**

Workers may experience higher HRI risk under rising AT, underscoring the need for worker-centered prevention and heat-risk communication in humid climates.

## 1 Introduction

Heatwaves are among the most consequential climate-related hazards, with increasing implications for population health and occupational safety. Epidemiologic evidence has long shown that heatwave periods are associated with elevated mortality, and that risk can vary meaningfully by event characteristics and population vulnerability (1). Looking forward, climate-change–driven warming—particularly the accumulation of heat across nights—may further increase heat-related mortality burdens, indicating that heat risk is not confined to isolated daytime extremes (2). In humid climates, the characterization of heat risk also depends critically on moisture: recent climate-science work emphasizes that humidity can materially affect how heat extremes are described and communicated, with direct relevance for human heat strain and operational risk messaging (3).

These issues are highly salient in East Asia, where large-scale circulation patterns contribute to extreme heatwave occurrence and persistence, including over Korea and neighboring regions (4). In South Korea specifically, human-perceived temperature metrics have shown notable changes over time and have been associated with heat-related health outcomes, supporting the use of “felt heat” indicators alongside conventional air temperature measures (5). Together, this body of evidence motivates occupationally relevant heat-risk assessment approaches that account for humid heat and identify risk escalation early enough to support prevention.

Workers engaged in outdoor or physically demanding tasks are particularly vulnerable to heat stress because metabolic heat production, work clothing or protective equipment, and limited access to cooling can compound environmental exposure. Core thermal-environment references and standards emphasize that heat strain emerges from the combined effects of environment, workload, and clothing, and that prevention requires practical decision support rather than reliance on a single meteorological metric (6–8). Operational guidance from occupational authorities similarly underscores early recognition of hazardous heat conditions and the use of preventive controls such as hydration, rest scheduling, acclimatization, and work modification (9–11). In practice, different indices are used to support these decisions. For example, wet-bulb globe temperature (WBGT)-based staging guidance is used in national heat-illness prevention information in Japan (12), and recent epidemiologic work has quantified associations between WBGT and heat-related illness outcomes, reinforcing the relevance of composite heat-stress indices for health protection (13).

Beyond heat illness, evidence indicates that occupational heat exposure contributes to broader safety impacts. A systematic review and meta-analysis reported consistent associations between extreme heat and occupational injuries across climate zones, supporting the interpretation that heat is both a clinical HRI issue and an occupational safety concern (14). Recent occupational studies have also examined severity patterns of heat-related injuries (15) and synthesized intervention strategies to prevent occupational heat stress, highlighting the value of actionable evidence for worksite prevention planning (16). In parallel, policy debates around heat protections—particularly for agriculture and construction—illustrate that occupational heat standards can become contested, increasing the importance of transparent, policy-relevant evidence (17).

Heat-health warning systems provide a key bridge between climate information and prevention actions. International guidance emphasizes that effective warning systems should align heat metrics and thresholds with health outcomes and local vulnerability patterns (18), and national experiences (e.g., France) demonstrate how warning system design and evaluation can evolve with expanding evidence and operational needs (19). Korea has strong enabling infrastructure for such evidence because a national emergency department–based surveillance system provides heat-related illness monitoring during summer seasons, including yearbook-style summaries and dataset documentation (20, 21). Coupled with nationwide meteorological observation data from the Korea Meteorological Administration (22), these resources enable a concise occupationally focused analysis even when employment status is not explicitly recorded in surveillance systems.

Accordingly, we leveraged national heat-related illness surveillance and meteorological records to (i) classify cases as occupationally related vs. non-occupational using a transparent rule-based algorithm applied to case circumstances, (ii) quantify and compare apparent temperature–HRI escalation patterns between groups, and (iii) interpret these patterns against commonly used apparent-temperature reference thresholds for heat-risk management.

## 2 Methods

### 2.1 Data sources

#### 2.1.1 HRI surveillance

Daily case-level HRI records were obtained from the Korea Disease Control and Prevention Agency (KDCA) heat-related illness emergency department–based surveillance system, which captures clinically diagnosed heat illnesses reported by participating emergency departments nationwide during summer operations (20). The corresponding dataset documentation and access information were also referenced for variable definitions and data handling (21). We restricted analyses to June–September, 2015–2024, consistent with the available dataset. HRI in this surveillance was defined using the Korean Standard Classification of Diseases, 8th revision (KCD-8) T67.x heat-related illness codes, including heat stroke (T67.0), heat syncope (T67.1), heat cramps (T67.2), heat exhaustion (T67.3–T67.5), heat edema (T67.7), and other effects of heat and light (T67.8–T67.9) (21).

### 2.2 Meteorological data

#### 2.2.1 Meteorological data and apparent temperature

Apparent temperature (AT) and related meteorological variables were obtained from the Korea Meteorological Administration (KMA) Open MET Data Portal for 16 major stations during Jun–Sep 2015–2024. Station-level AT was computed by the KMA algorithm that incorporates air temperature (Ta, °C) and relative humidity (RH, %) via an estimated wet-bulb temperature (Tw, °C). Specifically, Tw was approximated from Ta and RH using the empirical equation proposed by Roland Stull, and AT was then derived from Ta and Tw using the KMA summer AT equation (full equations provided in Supplementary Methods S1).

For exposure linkage, station-level daily AT values were aggregated to a national daily mean AT by averaging across the 16 stations for each date. To characterize spatial heterogeneity, we additionally summarized station-specific AT distributions and daily cross-station variability across the 16 stations (Supplementary Tables S1, S2).

### 2.3 Occupational vs. non-occupational classification

Because employment status is not explicitly recorded in the surveillance database, we used a transparent location-based classification algorithm. Occupational status was classified using a rule-based mapping of the KDCA occurrence location field. Full mapping rules and the complete list of location codes and labels used for classification are provided in Supplementary Methods S2 and Supplementary Table S3 (21). Because this approach infers work-relatedness from case circumstances rather than verified employment status, some misclassification is possible; we summarize prior use of this field and additional considerations (including the absence of linkage-based validation against worker registries in the available data) in Supplementary Note 1.

### 2.4 Expanded occupational definition (primary analysis)

Cases were classified as occupational if the recorded occurrence location indicated (i) indoor or outdoor workplaces or (ii) agricultural work settings, including fields/farms and greenhouses. All other locations were classified as non-occupational. This expanded definition was used as the primary analysis to better capture major work-related heat exposure settings while maintaining reproducible rules.

### 2.5 Sensitivity definition

As robustness checks, we conducted two sensitivity analyses. First, we applied a stricter occupational definition limited to explicit workplaces (indoor/outdoor workplaces only) (Supplementary Figure S1, Supplementary Table S4), acknowledging that agricultural settings may occasionally include non-work activities. Second, to assess robustness to geographic heterogeneity in AT, we repeated the main bin-based summaries and Poisson regression models using a priori inland vs. coastal station-group mean AT summaries (coastal: Busan, Incheon, Ulsan, Mokpo, and Jeju; inland: all remaining stations). In addition, where occurrence location fields were consistently populated, we performed an inland vs. coastal stratified sensitivity analysis using occurrence location, which was consistently populated from 2019 onward; therefore, this stratified analysis was restricted to 2019–2024 (Supplementary Tables S5, S6).

### 2.6 Statistical analysis

**Figure 1:**
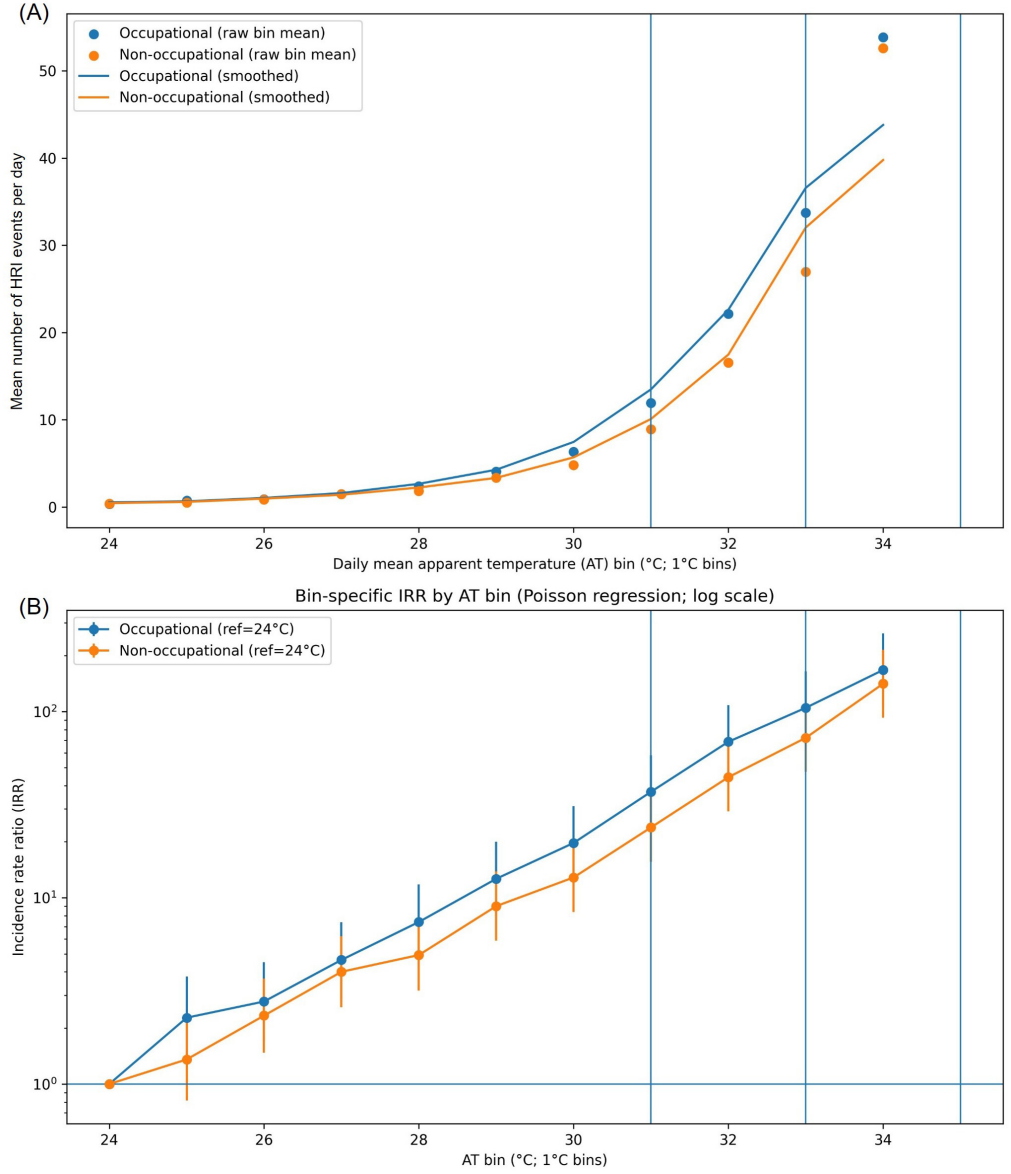
Apparent temperature (AT)–heat-related illness (HRI) relationships by occupational status in Korea, summers (Jun–Sep) 2015–2024. **(A)** Mean daily HRI events per day by 1 °C daily mean AT bins (24–34 °C). Points show raw bin means (mean daily HRI counts within each AT bin); lines show descriptive smoothing of the bin-wise means for visualization. **(B)** Bin-specific incidence rate ratios (IRRs) from Poisson regression (log scale), with the 24 °C bin as the reference (IRR = 1.0); vertical bars indicate 95% confidence intervals. Vertical guide lines mark commonly referenced AT thresholds (31 °C, 33 °C, 35 °C).

We linked daily national mean apparent temperature (AT) with daily occupational and non-occupational HRI counts for Jun–Sep 2015–2024. Daily AT was computed as the mean across 16 major stations. For bin-based summaries, we grouped days into 1 °C bins of daily mean AT (integer floor). “Bin-based summaries” refer to within-bin mean daily HRI counts for each series; these unsmoothed values are shown as points in Figure 1A and are referred to as “raw bin means.” To avoid unstable bin estimates, bins with fewer than 10 observation days were excluded. For bin-wise summaries and incidence rate ratios (IRR) estimation, analyses were restricted to AT bins 24–34 °C (1 °C bins; ≥10 days per bin), yielding *n* = 1,147 days (Table 1).

**Table 1:** Study overview and key design elements for the main analysis, including the KDCA emergency department–based heat-related illness (HRI) surveillance data source, study period (summers 2015–2024), analytic days, KCD-8 clinical case definition (T67.x), case counts, and the occupational vs. non-occupational classification.

**Item**	**Definition/value**
HRI surveillance source	KDCA emergency department–based heat-related illness (HRI) surveillance (case-level records) (20, 21)
HRI study window (overall)	Summers (June–September), 2015–2024
HRI clinical case definition (diagnostic scope)	Heat-related illness defined by KCD-8 codes T67.x, including heat stroke (T67.0), heat syncope (T67.1), heat cramps (T67.2), heat exhaustion (T67.3–T67.5), heat edema (T67.7), and other effects of heat and light (T67.8–T67.9) (21)
Clinical profile and severity context (KDCA 2024 summary)	In the 2024 KDCA ED surveillance summary (May 20–Sep 30), heat exhaustion accounted for 2,060/3,704 (55.6%) and heat stroke accounted for 732/3,704 (19.8%); heat stroke represented the predominant severe phenotype (20). Among 34 presumed heat-related deaths reported in 2024, 28 (82.4%) occurred outdoors and the presumed cause was predominantly heat stroke (94.1%) (20).
Meteorological source/metric	KMA station observations; apparent temperature (AT) (22)
Stations used	16 major stations; daily national mean AT computed as the mean across stations
Association analytic window (main analysis)	Summers (June–September), 2015–2024 (AT-bin analyses: AT bins 24–34 °C; 1 °C bins; ≥10 days/bin)
Analytic days (AT-bin analyses; 16-station mean; AT bins 24–34 °C; ≥10 days/bin)	*n* = 1,147 days
Total HRI cases (analytic days)	20,222
Occupational cases (primary definition: expanded)	11,112 (55.0%)
Non-occupational cases (primary definition: expanded)	9,110 (45.0%)
Primary occupational definition (expanded)	Classified as occupational if occurrence location indicated (i) indoor/outdoor workplaces or (ii) agricultural work settings (fields/farms, greenhouses); otherwise non-occupational
Sensitivity occupational definition (strict)	Classified as occupational only if occurrence location indicated indoor/outdoor workplaces (agricultural settings treated as non-occupational)

We estimated bin-specific IRRs (reference = 24 °C) and 95% confidence intervals using Poisson regression, separately for occupational and non-occupational series. Model estimates were reported for each AT bin relative to the 24 °C bin and visualized in Figure 1B. We selected 24 °C as the reference bin because it represents a moderate summer AT level with stable data density (≥10 days) and lies below the range where HRI counts begin to increase more noticeably, providing an interpretable baseline for relative risk comparisons across bins.

Geographic sensitivity analyses. To assess robustness to geographic heterogeneity in AT exposure summaries, we repeated the same binning and Poisson modeling framework using inland-mean vs. coastal-mean AT, computed from a priori station grouping (coastal: Busan, Incheon, Ulsan, Mokpo, and Jeju; inland: all remaining stations). Visualization (Figure 1A). We plotted mean daily HRI counts by 1 °C AT bin for occupational and non-occupational series. For descriptive smoothing of the plotted curves, we overlaid a centered 3-bin moving average (running mean) of the raw bin means to reduce small-sample noise across adjacent bins; this smoothing was used for visualization only and did not affect the Poisson IRR estimates in Figure 1B (23).

### 2.7 IRR estimation

We estimated IRRs by AT bin using Poisson regression with AT bin as a categorical predictor, separately for occupational and non-occupational daily counts. The 24 °C bin served as the reference category, and 95% confidence intervals were derived from model-based standard errors. We selected the 24 °C bin as the reference because it represents the lowest apparent-temperature category within the analytic window with sufficient observation days, providing a stable low-heat baseline for relative comparisons. For visual interpretation, we prespecified vertical reference lines at AT = 31 °C, 33 °C, and 35 °C in the figure; these lines were used as interpretive guides and were not used as thresholds within the statistical models. AT bins with fewer than 10 observation days were excluded from the IRR models to reduce coefficient instability.

### 2.8 Statistical software

All analyses and figures were generated in Python (pandas for data processing, statsmodels for Poisson regression, and matplotlib for plotting).

## 3 Results

### 3.1 Context: intensifying summer heat conditions in Korea

Across summers (June–September), Korea experienced a clear upward shift in both air temperature and apparent temperature (AT) over the past decade. In the 2015–2024 meteorological series, the nationwide mean summer temperature increased from 23.8 °C (2015) to 26.2 °C (2024), while mean summer AT increased from 28.2 °C to 30.6 °C. Consistent with this intensification, policy-relevant exceedance days based on daily maximum AT also increased substantially; for example, days with AT ≥ 31 °C rose from 24.5 days/city (2015) to 63.4 days/city (2024), and days with AT ≥ 33 °C rose from 9.4 to 33.8 days/city, indicating a marked expansion of high-heat conditions.

### 3.2 Study days and case counts

For AT-bin analyses (AT 24–34 °C; 1 °C bins), the dataset included *n* = 1,147 days and 20,222 HRI cases: 11,112 occupational (broad definition) and 9,110 non-occupational. Bin counts (#days), raw bin means, and IRRs for Figure 1 are summarized in Table 2. An equivalent bin-wise summary table for the strict occupational definition sensitivity analysis is provided in Supplementary Table S6. Given spatial variability in station-level AT, inland vs. coastal sensitivity analyses were additionally conducted and are presented in Supplementary Tables S5, S6 (Supplementary Figure S2).

**Table 2:** Bin-wise summaries for Figure 1 (broad occupational definition): mean daily heat-related illness (HRI) counts and Poisson regression incidence rate ratios (IRRs) by daily national mean apparent temperature (AT), June–September 2015–2024.

**AT bin (°C)**	**Days (*n*)**	**Occ mean/day**	**Non-occ mean/day**	**Occ IRR (95% CI)**	**Non-occ IRR (95% CI)**
24	59	0.32	0.37	1.00 (ref)	1.00 (ref)
25	93	0.73	0.51	2.27 (1.37–3.78)	1.36 (0.82–2.25)
26	122	0.89	0.87	2.77 (1.70–4.52)	2.33 (1.47–3.69)
27	152	1.49	1.49	4.64 (2.90–7.41)	4.01 (2.59–6.20)
28	129	2.39	1.84	7.41 (4.66–11.78)	4.93 (3.18–7.63)
29	124	4.07	3.36	12.65 (8.00–19.99)	9.02 (5.87–13.85)
30	122	6.34	4.80	19.70 (12.50–31.06)	12.86 (8.40–19.68)
31	98	11.94	8.90	37.07 (23.56–58.33)	23.86 (15.63–36.43)
32	107	22.16	16.56	68.81 (43.81–108.07)	44.41 (29.17–67.63)
33	101	33.73	26.94	104.75 (66.73–164.43)	72.25 (47.49–109.91)
34	40	53.85	52.60	167.22 (106.45–262.68)	141.06 (92.68–214.70)

Daily national mean AT was calculated as the mean apparent temperature across 16 major meteorological stations. Days were grouped into 1 °C AT bins (24–34 °C). For each bin, the table reports the number of analytic days, mean daily HRI counts for occupational and non-occupational cases, and IRRs (95% CI) from Poisson regression using the 24 °C bin as the reference.

### 3.3 Apparent temperature variability across stations

Across the 16 stations on the analytic days used for Figure 1 (*n* = 1,147), mean station-level AT ranged from 28.11 °C (Incheon) to 30.26 °C (Gwangju), and inland stations were, on average, warmer than coastal stations (mean difference: 0.55 °C; Supplementary Table S2). At the day level, the median across-station standard deviation was 1.28 °C (95th percentile: 2.16 °C) and the median across-station range was 4.70 °C (95th percentile: 7.50 °C). These findings suggest non-trivial spatial variability in AT across stations; therefore, we present inland–coastal analyses as a supplementary sensitivity check.

### 3.4 Differential AT–HRI escalation by occupational status

Bin-wise patterns (Figure 1A). Mean daily HRI counts increased with higher AT in both groups. In Figure 1A, points represent raw bin-wise means and lines represent a simple 3-bin centered rolling mean for visualization. Occupational raw bin means were generally higher than non-occupational means across bins (equal at 27 °C and slightly lower at 24 °C) and diverged more strongly at warmer AT bins. For example, occupational mean counts rose from 0.32/day at 24 °C to 11.94/day at 31 °C, 33.73/day at 33 °C, and 53.85/day at 34 °C; corresponding non-occupational means were 0.37/day, 8.90/day, 26.94/day, and 52.60/day.

IRRs by AT bin (Figure 1B). In Poisson models using the 24 °C bin as the reference, IRRs increased monotonically with AT for both series, and occupational IRRs tended to be higher than non-occupational IRRs at warmer bins. Key comparisons at commonly referenced heat-impact levels were:

31 °C: occupational IRR 37.07 (95% CI 23.56–58.33) vs. non-occupational IRR 23.86 (95% CI 15.63–36.43)

33 °C: occupational IRR 104.75 (95% CI 66.73–164.43) vs. non-occupational IRR 72.25 (95% CI 47.49–109.91)

34 °C: occupational IRR 167.22 (95% CI 106.45–262.68) vs. non-occupational IRR 141.06 (95% CI 92.68–214.70)

Across the observed AT range, occupational risk estimates were generally higher than non-occupational estimates at warmer bins, although uncertainty intervals overlap at some bins. Overall, these findings are consistent with the need for worker-centered prevention strategies under humid heat.

### 3.5 Sensitivity analyses

Sensitivity analyses using a strict occupational definition that classifies only explicit indoor/outdoor workplaces as occupational (treating agricultural settings as non-occupational) showed similar IRR patterns, indicating that the primary conclusions are robust to the occupational case definition (Supplementary Figure S1; Supplementary Table S4). In an additional geographic sensitivity analysis stratifying cases by inland vs. coastal occurrence location (2019–2024) and summarizing exposure using inland-mean and coastal-mean AT, bin-wise IRR patterns remained monotonic and occupational IRRs generally exceeded non-occupational IRRs in both strata (Supplementary Tables S5, S6). Because occurrence location was not consistently populated before 2019, inland–coastal stratified results are reported for 2019–2024; we also repeated the primary national analysis for 2019–2024 and observed consistent bin-wise patterns (Supplementary Tables S7, S8).

## 4 Discussion

### 4.1 Principal findings and interpretation

Using national emergency department–based HRI surveillance linked with nationwide meteorological apparent temperature (AT), we found that HRI risk increased non-linearly with AT in both occupational and non-occupational series when exposure was summarized as national mean daily AT (Figure 1). At warmer AT bins, occupational IRR estimates tended to be higher than non-occupational estimates (Figure 1; Table 2). In bin-specific comparisons using 24 °C as the reference, occupational IRRs became statistically elevated starting at 25 °C, whereas non-occupational IRRs became elevated starting at 26 °C (Table 2). However, bin-specific confidence intervals overlapped at several AT bins (Table 2). Accordingly, apparent differences in the “onset” range and the relative pace of escalation should be interpreted cautiously as descriptive patterns rather than statistically distinct thresholds separating the two groups. The overall directionality was robust to an alternative occupational definition (Supplementary Figure S1; Supplementary Table S4) and to geographic sensitivity analyses (Supplementary Tables S5, S6), supporting the practical implication that worker-centered prevention may need to begin under moderate-to-high AT conditions, not only during extreme heat.

Importantly, the surveillance case definition encompasses a spectrum of clinically diagnosed heat illnesses (KCD-8 T67.x), including heat exhaustion and heat stroke (21). Although heat exhaustion constitutes the majority of cases in KDCA summaries, heat stroke represents a severe phenotype and predominates among presumed heat-related deaths. In the 2024 KDCA summary, heat exhaustion accounted for 55.6% of cases while heat stroke accounted for 19.8% and predominated among severe outcomes, including 94.1% of presumed heat-related deaths (Table 1) (20). This clinical profile underscores the public health relevance of rising risk under warmer AT: even modest shifts in risk across commonly experienced temperature ranges can translate into meaningful increases in case burden and potentially severe outcomes during hot seasons.

### 4.2 Relation to prior evidence and plausible mechanisms

Our findings align with occupational heat-stress frameworks emphasizing that heat strain reflects the combined effects of environment, metabolic workload, clothing, and behavioral constraints, and that prevention should rely on early, practical controls rather than waiting for extreme thresholds (6–8). Occupational guidance commonly highlights hydration, rest scheduling, acclimatization, and work modification as foundational controls (9–11). The tendency toward higher occupational risk under warmer AT is consistent with these frameworks: workers may have limited ability to self-pace or relocate, and internal heat production and protective equipment can amplify physiologic strain relative to non-occupational populations under similar ambient conditions (6–8).

The occupational contrast also agrees with literature linking heat exposure to occupational harms beyond classic heat illness, including occupational injuries (14, 15). A meta-analysis reported consistent associations between extreme heat and occupational injuries across climate zones (14), and recent occupational studies have examined severity patterns and intervention strategies for heat-related injuries and heat stress (15, 16). Although our outcome is HRI rather than injury, the convergence of evidence supports a coherent interpretation: heat elevates multiple dimensions of occupational risk, and early-stage increases across moderate-to-high heat ranges are meaningful for prevention planning.

### 4.3 Policy relevance and measurement considerations

In Korea and other humid settings, AT is a policy-relevant communication metric because humidity can materially shape perceived heat and physiologic strain (3), and human-perceived heat indicators have shown notable temporal and spatial patterns (5). Our bin-based IRR presentation was designed to be decision-friendly, offering a clear view of how risk changes across AT ranges while directly contrasting occupational vs. non-occupational series. This approach complements heat–health warning principles that emphasize aligning warning triggers with health outcomes and vulnerability patterns (18).

At the same time, the vertical reference lines at 31/33/35 °C in Figure 1 are interpretive guides rather than operational thresholds, because operational criteria often rely on local forecasts and daily maxima, whereas we used national mean daily AT to construct a consistent nationwide exposure metric. Even under this conservative exposure summary, occupational risk estimates tended to be higher at warmer AT bins, reinforcing the practical inference that workplace heat-risk management should not rely solely on extreme-heat thresholds.

### 4.4 Strengths and limitations

Strengths of this brief analysis include the use of a nationwide, clinically based surveillance system (20, 21), a transparent and reproducible occupational classification algorithm, and a policy-readable IRR framework that contrasts occupational vs. non-occupational patterns across AT bins. In addition, restricting the analysis to complete-case days with high station completeness supports consistent aggregation of nationwide apparent temperature (AT) exposure (22), and we summarized cross-station AT variability to contextualize geographic sensitivity analyses (Supplementary Tables S1, S2).

Several limitations warrant mention. First, this is an ecological analysis using aggregated AT summaries, which may dilute local extremes and cannot represent individual exposure, workload, clothing, microenvironments, or access to cooling. Second, occupational classification is inferred from occurrence location rather than verified employment status, and we could not validate this algorithm against linkage to worker registries within the available data; some misclassification is therefore possible and should be considered when interpreting occupational–non-occupational contrasts. We addressed this concern in part through a strict-definition sensitivity analysis restricted to explicit workplace locations (Supplementary Figure S1; Supplementary Table S4) and through additional discussion of classification considerations (Supplementary Note 1). Third, although occupational point estimates were generally higher at warmer bins, bin-specific confidence intervals overlapped at several AT levels, limiting inference about statistically distinct “onset” thresholds or systematically steeper escalation between groups based solely on bin-wise comparisons (Table 2). Fourth, emergency department surveillance may under-ascertain mild cases and is subject to reporting heterogeneity, though its national scale and standardized summer operation support its utility for comparative risk patterns (20, 21). Finally, geographic stratified sensitivity analyses relied on occurrence-location fields and were restricted to 2019–2024, when these fields were consistently populated; reassuringly, repeating the primary national analysis within 2019–2024 yielded consistent patterns (Supplementary Tables S7, S8).

## 5 Conclusion

In summary, national ED-based HRI surveillance linked with nationwide apparent temperature indicates that HRI risk rises substantially with warmer AT in both occupational and non-occupational series. Under a national-mean exposure summary, occupational IRR estimates tended to be higher than non-occupational estimates at warmer AT bins, with bin-specific comparisons suggesting statistical elevation beginning at 25 °C for occupational cases and 26 °C for non-occupational cases (Table 2). Because bin-specific uncertainty overlaps at several AT levels, differences in the apparent “onset” range and escalation should be interpreted cautiously as descriptive patterns rather than statistically distinct thresholds. Nonetheless, the consistent directionality across alternative occupational definitions and geographic sensitivity analyses supports the practical value of worker-centered prevention strategies that activate during moderate-to-high AT conditions, rather than waiting for extreme-heat thresholds. This brief, policy-aligned evidence can help refine heat-risk communication and workplace heat-management decisions in humid, mid-latitude settings experiencing intensifying summer heat conditions.

## Data Availability

The raw data supporting the conclusions of this article will be made available by the authors, without undue reservation.
