# Supplementary material for: Consistently higher and steeper apparent temperature–heat-related illness risk among occupational cases in Korea: evidence from national emergency department surveillance
Source: Front Public Health. 2026 Mar 11;14:1786564. doi: 10.3389/fpubh.2026.1786564 (PMC13013529; doi:10.3389/fpubh.2026.1786564)
Supplement: Supplementary file 1 [file Supplementary_file_1.docx]

Supplementary Material

**Supplementary Table S1**. Station-specific apparent temperature (AT) summaries across 16 stations (Jun–Sep 2015–2024).

| Station | Group | Mean AT (°C) | SD AT (°C) | Min AT (°C) | Max AT (°C) | N days |
| --- | --- | --- | --- | --- | --- | --- |
| Jeju | Coastal | 29.73 | 3.17 | 21.30 | 36.30 | 1147 |
| Mokpo | Coastal | 29.22 | 2.92 | 22.60 | 35.70 | 1147 |
| Ulsan | Coastal | 28.89 | 3.20 | 20.80 | 35.70 | 1147 |
| Busan | Coastal | 28.73 | 2.94 | 21.40 | 36.20 | 1147 |
| Incheon | Coastal | 28.11 | 2.98 | 20.10 | 35.30 | 1147 |
| Gwangju | Inland | 30.26 | 2.91 | 23.40 | 37.40 | 1147 |
| Jeonju | Inland | 30.06 | 2.94 | 22.70 | 37.20 | 1147 |
| Gimhae | Inland | 30.04 | 3.09 | 21.80 | 37.00 | 1147 |
| Daejeon | Inland | 29.81 | 2.96 | 21.40 | 36.80 | 1147 |
| Gyeongju | Inland | 29.67 | 3.53 | 20.50 | 38.00 | 1141 |
| Daegu | Inland | 29.51 | 3.24 | 22.00 | 37.00 | 1147 |
| Suwon | Inland | 29.47 | 2.92 | 22.10 | 36.70 | 1147 |
| Cheongju | Inland | 29.31 | 3.04 | 22.10 | 35.80 | 1147 |
| Seoul | Inland | 29.06 | 2.89 | 20.30 | 35.70 | 1147 |
| Cheonan | Inland | 28.72 | 2.92 | 20.90 | 34.80 | 1147 |
| Wonju | Inland | 28.43 | 2.97 | 21.40 | 35.40 | 1147 |

**Supplementary Table S2**. Daily cross-station AT variability across the 16 stations (Jun–Sep 2015–2024; analytic days with AT bins 24–34°C).

| Metric | Mean | Median | P25 | P75 | P95 |
| --- | --- | --- | --- | --- | --- |
| Across-station SD (°C) | 1.342 | 1.285 | 1.026 | 1.574 | 2.159 |
| Across-station range (°C) | 4.810 | 4.700 | 3.700 | 5.700 | 7.500 |
| Inland minus Coastal mean (°C) | 0.549 | 0.544 | -0.126 | 1.256 | 2.302 |

**Supplementary Table S3**. Classification of occupational status based on KDCA occurrence locations. categorical occurrence location values from the KDCA surveillance system and their corresponding occupational classifications under the expanded (primary) and strict (sensitivity) definitions [21].

| **KDCA occurrence location (Korean)** | **Suggested English label** | **Expanded definition (primary)** | **Strict definition (sensitivity)** |
| --- | --- | --- | --- |
| 실외 작업장 | Outdoor workplace | Occupational | Occupational |
| 실내 작업장 | Indoor workplace | Occupational | Occupational |
| 논밭 | Field/Farm | Occupational | Non-occupational |
| 비닐하우스 | Greenhouse | Occupational | Non-occupational |
| 길가 | Roadside/Street | Non-occupational | Non-occupational |
| 집 | Home | Non-occupational | Non-occupational |
| 주거지 주변 | Around residence | Non-occupational | Non-occupational |
| 운동장(공원) | Park/Sports ground | Non-occupational | Non-occupational |
| 산 | Mountain | Non-occupational | Non-occupational |
| 강가,해변 | Riverside/Beach | Non-occupational | Non-occupational |
| 건물 | Building (non-work specified) | Non-occupational | Non-occupational |
| 실외 기타 | Other outdoor | Non-occupational | Non-occupational |
| 실내 기타 | Other indoor | Non-occupational | Non-occupational |

**Supplementary Table S4**. Bin-wise summary table for Supplementary Figure S1 (strict occupational definition; national mean AT).

| AT bin (°C) | Days (n) | Occ mean/day | Non-occ mean/day | Occ IRR (95% CI) | Non-occ IRR (95% CI) |
| --- | --- | --- | --- | --- | --- |
| 24 | 59 | 0.17 | 0.53 | 1.00 (ref) | 1.00 (ref) |
| 25 | 93 | 0.37 | 0.87 | 2.16 (1.07–4.37) | 1.66 (1.10–2.51) |
| 26 | 122 | 0.55 | 1.2 | 3.24 (1.67–6.30) | 2.29 (1.56–3.38) |
| 27 | 152 | 0.82 | 2.16 | 4.81 (2.53–9.17) | 4.11 (2.84–5.94) |
| 28 | 129 | 1.53 | 2.68 | 9.06 (4.80–17.09) | 5.10 (3.54–7.37) |
| 29 | 124 | 2.56 | 4.83 | 15.08 (8.04–28.31) | 9.19 (6.41–13.19) |
| 30 | 122 | 4.35 | 6.73 | 25.68 (13.74–48.00) | 12.81 (8.95–18.33) |
| 31 | 98 | 8.62 | 12.06 | 50.87 (27.27–94.89) | 22.96 (16.07–32.79) |
| 32 | 107 | 16.14 | 22.28 | 95.23 (51.15–177.30) | 42.40 (29.75–60.43) |
| 33 | 101 | 24.32 | 36.01 | 143.47 (77.10–266.98) | 68.53 (48.13–97.60) |
| 34 | 40 | 38.95 | 67.15 | 229.80 (123.40–427.95) | 127.80 (89.70–182.09) |

**Supplementary Table S5**. Inland vs coastal stratified sensitivity (2019–2024): inland stratum (cases occurring in inland regions; inland-mean AT). Note: Province/county occurrence fields are not populated in 2015–2018 in this surveillance release; therefore, stratified analyses are restricted to 2019–2024.

| AT bin (°C) | Days (n) | Occ mean/day | Non-occ mean/day | Occ IRR (95% CI) | Non-occ IRR (95% CI) |
| --- | --- | --- | --- | --- | --- |
| 24 | 31 | 0.23 | 0.26 | 1.00 (ref) | 1.00 (ref) |
| 25 | 54 | 0.39 | 0.35 | 1.72 (0.73–4.05) | 1.36 (0.60–3.11) |
| 26 | 53 | 0.89 | 0.75 | 3.93 (1.78–8.69) | 2.92 (1.37–6.25) |
| 27 | 79 | 0.8 | 0.82 | 3.53 (1.62–7.71) | 3.19 (1.53–6.64) |
| 28 | 84 | 1.6 | 1.62 | 7.06 (3.30–15.10) | 6.27 (3.08–12.80) |
| 29 | 87 | 3.16 | 2.45 | 14.00 (6.61–29.64) | 9.49 (4.68–19.22) |
| 30 | 84 | 4.49 | 3.63 | 19.88 (9.41–41.98) | 14.07 (6.97–28.39) |
| 31 | 72 | 7.04 | 5.96 | 31.18 (14.79–65.75) | 23.09 (11.47–46.47) |
| 32 | 57 | 15.19 | 10.35 | 67.28 (31.98–141.56) | 40.11 (19.97–80.58) |
| 33 | 71 | 22.92 | 16.54 | 101.48 (48.30–213.21) | 64.07 (31.97–128.43) |
| 34 | 29 | 38.28 | 29.34 | 169.51 (80.62–356.39) | 113.71 (56.68–228.12) |

**Supplementary Table S6**. Inland vs coastal stratified sensitivity (2019–2024): coastal stratum (cases occurring in coastal regions; coastal-mean AT). Note: Province/county occurrence fields are not populated in 2015–2018 in this surveillance release; therefore, stratified analyses are restricted to 2019–2024.

| AT bin (°C) | Days (n) | Occ mean/day | Non-occ mean/day | Occ IRR (95% CI) | Non-occ IRR (95% CI) |
| --- | --- | --- | --- | --- | --- |
| 24 | 41 | 0.2 | 0.17 | 1.00 (ref) | 1.00 (ref) |
| 25 | 53 | 0.32 | 0.25 | 1.64 (0.71–3.81) | 1.44 (0.57–3.60) |
| 26 | 82 | 0.56 | 0.33 | 2.87 (1.36–6.09) | 1.93 (0.84–4.43) |
| 27 | 85 | 0.54 | 0.32 | 2.77 (1.31–5.88) | 1.86 (0.81–4.27) |
| 28 | 76 | 0.79 | 0.49 | 4.05 (1.93–8.46) | 2.85 (1.27–6.40) |
| 29 | 77 | 1.31 | 0.88 | 6.72 (3.27–13.81) | 5.17 (2.38–11.26) |
| 30 | 79 | 2.53 | 1.13 | 12.97 (6.40–26.30) | 6.60 (3.06–14.24) |
| 31 | 75 | 4.45 | 2.63 | 22.82 (11.32–46.02) | 15.38 (7.24–32.69) |
| 32 | 71 | 7.46 | 4.48 | 38.26 (19.03–76.90) | 26.23 (12.41–55.48) |
| 33 | 43 | 10.28 | 7.19 | 52.68 (26.18–106.00) | 42.09 (19.90–89.03) |

**Supplementary Table S7**. Bin-wise summary table for the main analysis restricted to 2019–2024 (broad occupational definition; national mean AT) to match the inland/coastal stratified sensitivity analysis (occurrence-location completeness).

| AT bin (°C) | Days (n) | Occ mean/day | Non-occ mean/day | Occ IRR (95% CI) | Non-occ IRR (95% CI) |
| --- | --- | --- | --- | --- | --- |
| 24 | 30 | 0.33 | 0.43 | 1.00 (ref) | 1.00 (ref) |
| 25 | 49 | 0.78 | 0.47 | 2.33 (1.16–4.67) | 1.08 (0.55–2.14) |
| 26 | 61 | 1.18 | 1.10 | 3.54 (1.83–6.86) | 2.53 (1.40–4.59) |
| 27 | 95 | 1.47 | 1.44 | 4.42 (2.33–8.40) | 3.33 (1.88–5.88) |
| 28 | 80 | 2.95 | 2.11 | 8.85 (4.70–16.66) | 4.87 (2.77–8.57) |
| 29 | 86 | 4.60 | 3.71 | 13.81 (7.38–25.87) | 8.56 (4.92–14.90) |
| 30 | 82 | 7.04 | 4.99 | 21.11 (11.30–39.44) | 11.51 (6.63–19.99) |
| 31 | 62 | 11.69 | 8.50 | 35.08 (18.80–65.48) | 19.62 (11.31–34.01) |
| 32 | 70 | 22.74 | 15.29 | 68.23 (36.64–127.05) | 35.27 (20.42–60.95) |
| 33 | 63 | 35.78 | 23.79 | 107.33 (57.67–199.76) | 54.91 (31.81–94.79) |
| 34 | 17 | 51.53 | 46.41 | 154.59 (82.88–288.32) | 107.10 (61.91–185.28) |

**Supplementary Table S8**. Bin-wise summary table for the main analysis restricted to 2019–2024 (strict occupational definition; national mean AT) to match the inland/coastal stratified sensitivity analysis (occurrence-location completeness).

| AT bin (°C) | Days (n) | Occ mean/day | Non-occ mean/day | Occ IRR (95% CI) | Non-occ IRR (95% CI) |
| --- | --- | --- | --- | --- | --- |
| 24 | 30 | 0.20 | 0.57 | 1.00 (ref) | 1.00 (ref) |
| 25 | 49 | 0.45 | 0.80 | 2.24 (0.91–5.54) | 1.40 (0.79–2.48) |
| 26 | 61 | 0.89 | 1.39 | 4.43 (1.90–10.29) | 2.46 (1.46–4.14) |
| 27 | 95 | 0.81 | 2.11 | 4.05 (1.77–9.30) | 3.72 (2.26–6.10) |
| 28 | 80 | 2.00 | 3.06 | 10.00 (4.43–22.59) | 5.40 (3.31–8.84) |
| 29 | 86 | 3.03 | 5.28 | 15.17 (6.76–34.09) | 9.32 (5.74–15.12) |
| 30 | 82 | 5.02 | 7.00 | 25.12 (11.22–56.24) | 12.35 (7.63–20.01) |
| 31 | 62 | 8.50 | 11.69 | 42.50 (19.01–95.03) | 20.64 (12.76–33.38) |
| 32 | 70 | 17.23 | 20.80 | 86.14 (38.62–192.12) | 36.71 (22.76–59.21) |
| 33 | 63 | 26.44 | 33.13 | 132.22 (59.32–294.73) | 58.46 (36.27–94.22) |
| 34 | 17 | 37.12 | 60.82 | 185.59 (83.06–414.67) | 107.34 (66.47–173.33) |

**Supplementary Method S1. Apparent temperature calculation**

To calculate apparent temperature (AT), wet-bulb temperature (Tw) was first estimated from air temperature and relative humidity using the empirical formulation shown in Equation (1).

Reference: Stull R. Wet-Bulb Temperature from Relative Humidity and Air Temperature. J Appl Meteor Climatol. 2011.

Equation (1) :

Tw = Ta·atan[0.151977(RH+8.313659)¹ᐟ²] + atan(Ta+RH) − atan(RH−1.67633) + 0.00391838·RH³ᐟ²·atan(0.023101·RH) − 4.686035

where Tₐ is air temperature (°C) and RH is relative humidity (%).

AT was then derived from air temperature and wet-bulb temperature as follows:

Equation (2) : AT = -0.2442 + 0.55399Tw + 0.45535Ta – 0.0022Tw^2^ + 0.00278TwTa + 3.0

where Tₐ is air temperature (°C) and Tw is wet-bulb temperature estimated using Equation (1).

## Supplementary Methods S2. Occupational classification algorithm (expanded vs strict)

Because explicit employment status is not recorded in the KDCA emergency department–based HRI surveillance dataset, occupational status was classified using a **transparent, rule-based algorithm** based on the categorical “occurrence location” field recorded at the time of emergency department reporting [21]. We first created a three-level occupational plausibility variable and then collapsed it into the two binary definitions used in the main and sensitivity analyses.

### S2.1. Three-level occupational plausibility

Using the KDCA occurrence location categories, cases were assigned to:

- **Occupational (definite):** explicit workplaces
- **Occupational (probable):** agricultural work settings
- **Non-occupational:** all other settings

### S2.2. Binary occupational definitions used in analyses

- **Expanded occupational definition (primary analysis):**
  **Occupational = definite + probable** (explicit workplaces + agricultural work settings).
  This definition was selected to better capture major work-related heat exposure settings in Korea, particularly agriculture.
- **Strict occupational definition (sensitivity analysis):**
  **Occupational = definite only** (explicit workplaces only).
  This definition was used to assess robustness to potential ambiguity in agricultural settings.

**Supplementary Note 1. Misclassification considerations**

Some misclassification is possible because “occurrence location” may not perfectly capture employment status (e.g., non-workers in agricultural settings or workers in non-explicit locations). However, such misclassification is expected to be largely **non-differential with respect to national mean AT,** which would generally **bias occupational–non-occupational contrasts toward the null** rather than inflate observed differences. The strict-definition sensitivity analysis (Supplementary Figure S1) addresses this concern by restricting occupational classification to explicit workplaces only.


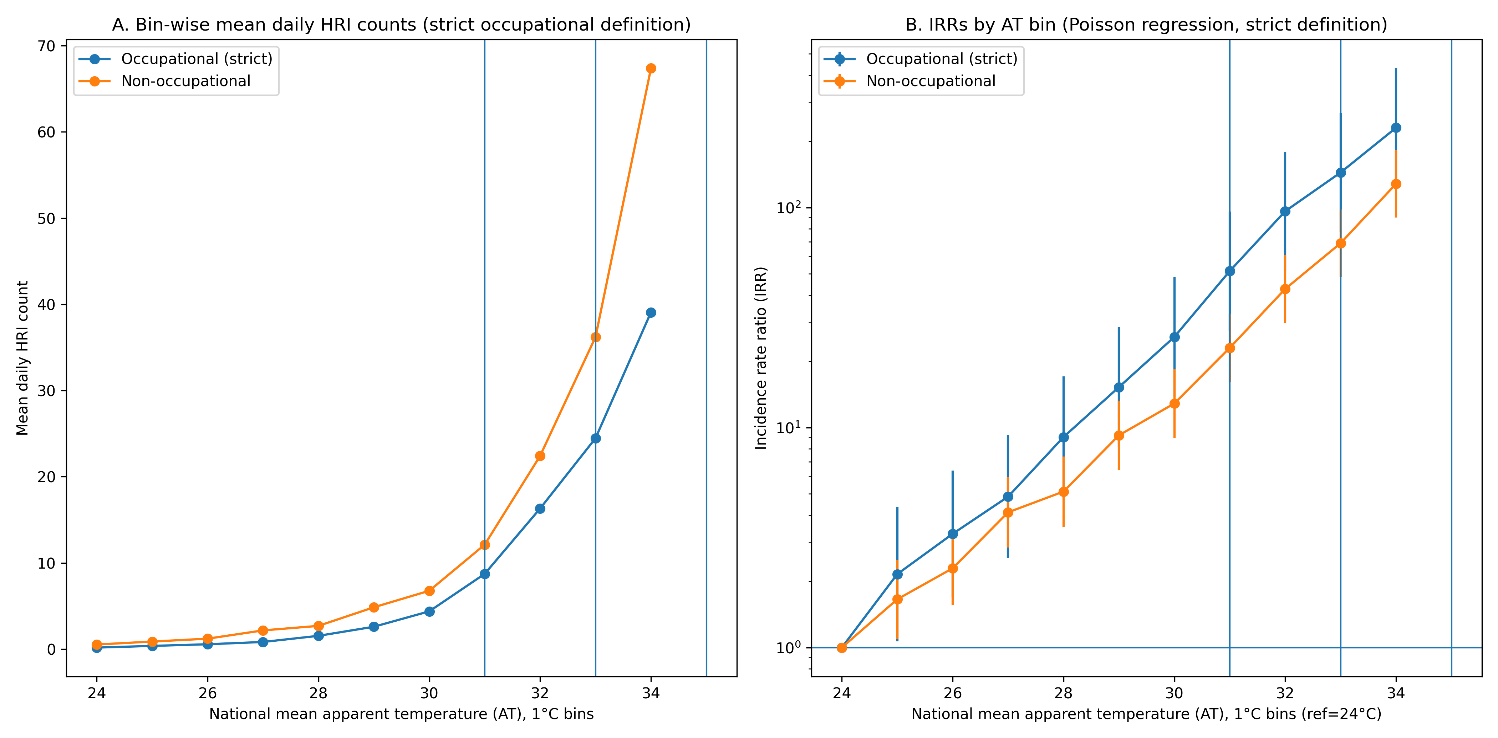


**Supplementary Figure S1. Sensitivity analysis using the strict occupational definition:**
(A) Bin-wise mean daily HRI counts by 1°C bins of national mean apparent temperature (AT). (B) Incidence rate ratios (IRRs) by AT bin from Poisson regression (reference = 24°C), shown on a log scale. The strict definition classifies cases as occupational only when occurrence location indicates explicit indoor/outdoor workplaces; agricultural settings are treated as non-occupational (see Supplementary Methods S1). Vertical reference lines at AT = 31°C, 33°C, and 35°C are shown for interpretive context. Under the strict definition, IRRs were already elevated at moderate AT bins (occupational IRR > 1 from 25–26°C), and occupational IRRs remained higher at warmer AT bins, indicating that the primary conclusion is not driven solely by inclusion of agricultural settings.


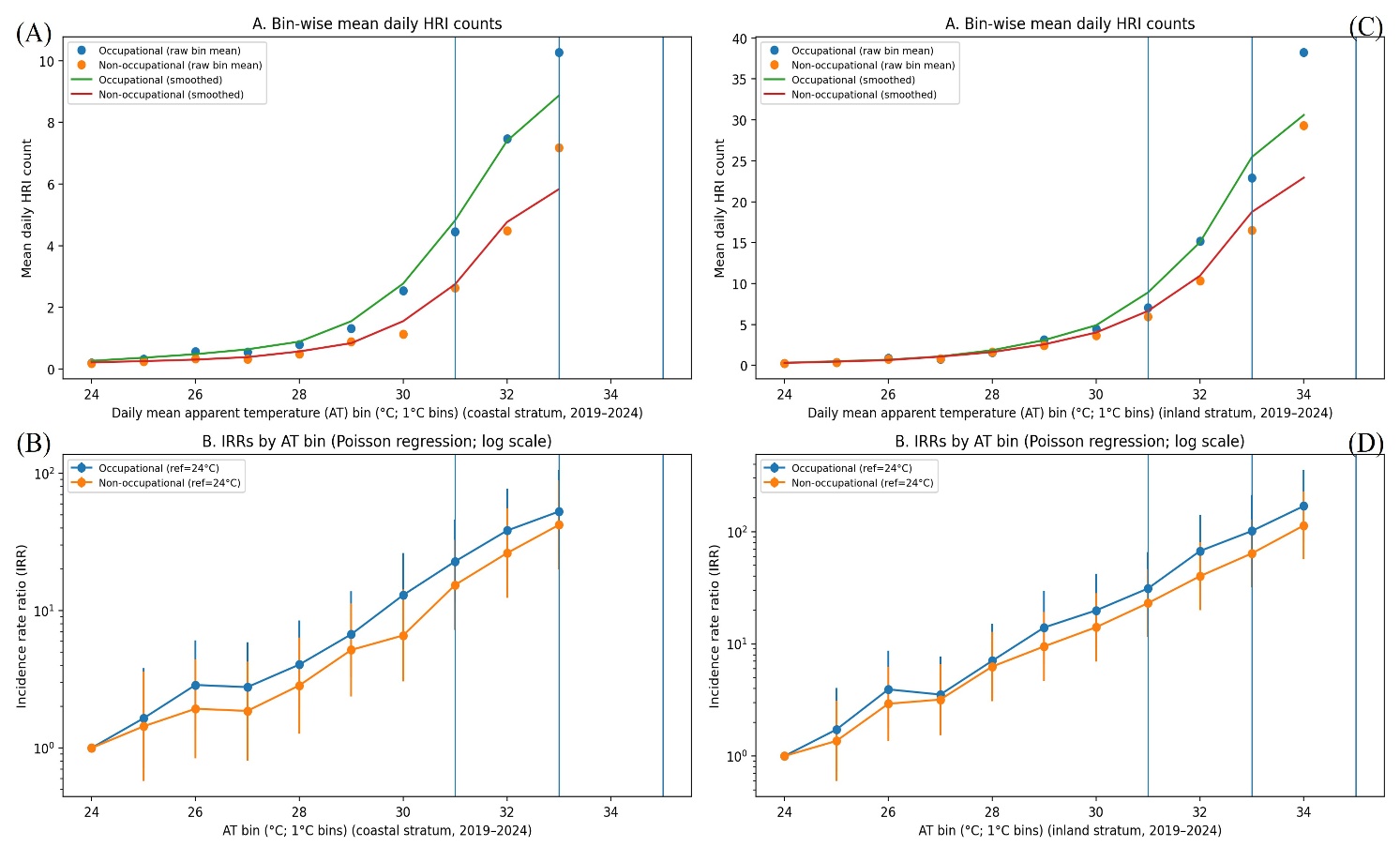


**Supplementary Figure S2. Inland–coastal stratified sensitivity analysis (2019–2024)**: bin-wise mean daily HRI counts and bin-specific incidence rate ratios (IRRs) by apparent temperature (AT).
Panels A–B show the coastal stratum, and panels C–D show the inland stratum (stratified using occurrence location; restricted to 2019–2024 when the occurrence location field was consistently populated). Panels A and C present bin-wise mean daily HRI counts, where points denote raw bin means and lines show descriptive smoothing using a centered 3-bin moving average (running mean). Panels B and D present bin-specific IRRs (reference = 24°C bin) estimated from Poisson regression, plotted on a log scale with 95% confidence intervals. Vertical reference lines denote AT = 31, 33, and 35°C.
